# Predictive value of clinical toxicities of chemotherapy with fluoropyrimidines and oxaliplatin in colorectal cancer by *DPYD* and *GSTP1* gene polymorphisms

**DOI:** 10.1186/s12957-020-02103-3

**Published:** 2020-12-06

**Authors:** Xunwei Deng, Jingyuan Hou, Qiaoting Deng, Zhixiong Zhong

**Affiliations:** 1 Department of Research Experimental Center, Meizhou People’s Hospital (Huangtang Hospital), Meizhou Hospital Affiliated to Sun Yat-Sen University, No. 63 Huangtang Road, Meijiang District, Meizhou, 514031 People’s Republic of China; 2 Guangdong Provincial Key Laboratory of Precision Medicine and Clinical Translational Research of Hakka Population, Meizhou, People’s Republic of China

**Keywords:** *DPYD*, *GSTP1*, Gene polymorphism, Chemotherapy toxicity, Hakka population

## Abstract

**Background:**

Fluoropyrimidines and platinum are still widely used for colorectal cancer (CRC) management. Several studies have reported that mutations of dihydropyrimidine dehydrogenase (*DPYD*) and glutathione S-transferase pi-1 (*GSTP1*) polymorphisms are related to chemotherapy-related adverse events. In the present study, we purposed to assess the impact of *DPYD* and *GSTP1* variants on the toxicity of adjuvant chemotherapy risk among the Hakka population, minimize adverse events, and to maximize therapy outcome for individualized treatment.

**Methods:**

Genotyping was examined in 104 patients diagnosed with CRC cases and receiving fluoropyrimidine and platinum drug-based chemotherapy regimen by direct sequencing of *DPYD* and *GSTP1* polymorphisms. Three *DPYD* variants including *2A, *5A, *9A, and *GSTP1* c.313A>G were analyzed and clinical outcomes were assessed.

**Results:**

The data suggest that the incidence of DPYD*5A, DPYD*9A, and *GSTP1* c.313A>G variants were 38.4%, 24%, and 32.7%, respectively. DPYD*2A variant was not found. A total of 23 patients (22.1%) suffered severe vomiting and 19 patients (18.3%) suffered severe anemia. DPYD*5A polymorphism was found significantly associated with grade 3/4 ulceration (*p* = 0.001). *GSTP1* was determined to be an independent risk factor for severe vomiting and skin ulceration (*p* = 0.042 and *p* = 0.018, respectively). Patients with *GSTP1* c. 313A>G mutant type contributed to a higher risk for grade severe toxicity compared with wild genotype (*p* = 0.027). Nevertheless, no significant difference was found between patients with DPYD*2A, *5A, and *9A for chemotherapeutic toxicity.

**Conclusions:**

The results demonstrated that *GSTP1* polymorphisms were useful predictors of severe events. Screening of single-nucleotide polymorphisms of *GSTP1* in colorectal cancer patients before chemotherapy may help to realize personalized therapy.

## Background

To date, the malignant tumor has become responsible for the majority of global deaths, and its incidence has been increasing over recent decades [1]. For both sexes combined, CRC is the third most prevalent diagnosed gastrointestinal malignancies globally and remains the fifth common cause of mortality by cancer in China, which is responsible for more than 191,000 deaths annually [1, 2].

5-FU and capecitabine, an oral prodrug of 5-FU, are the backbone of the therapeutic scheme for CRC and many other solid tumors including monotherapy and combined with other chemotherapy drugs. Common combination regimens include FOLFOX [3]: (oxaliplatin combined with bolus/infusional 5-FU and leucovorin) and XELOX [4]: (capecitabine plus oxaliplatin). Unfortunately, approximately 10 to 30% of patients display varying degrees of adverse effects [5], most frequent manifestations include anemia, neutropenia, nausea, vomiting, diarrhea, and neurological toxicities. Various clinical studies revealed that dihydropyrimidine dehydrogenase (DPD), encoded by the *DPYD* gene, is the limiting velocity enzyme in the catabolism of fluoropyrimidines. DPD is responsible for catabolism, more than 80% of given fluoropyrimidines. Deficiency of the DPYD gene results in excess fluoropyrimidine accumulation in the blood and increases the risk of toxicity. DPD enzyme activity detection currently commonly used methods are western blotting and HPLC [6, 7]. Western blotting requires preliminary experiments to determine the best conditions, and there are many interference factors. HPLC is expensive and not widely available in hospitals. Numerous studies have revealed that the polymorphism of the *DPYD* gene mutation results in a significantly decreased enzyme activity, such as well-known IVS14+1G>A (DPYD*2A). The *DPYD* IVS14+1G>A alternate splicing in intron 14 resulting in creating a fragmentary protein and leading to the lack of normal DPD enzyme function. *DPYD* c. 1627A>G (DPYD*5A) and *DPYD* c. 85T>C (DPYD*9A) are linked with decreased enzyme activity. In codon 29 of DPYD at the 85 position, the point mutation of T to C gives rise to an amino acid alteration from Cys to Arg, and *DPYD* 1627A>G substitution results in the DPYD*5A allele due to the change in I543V. In different ethnic groups, *DPYD* genetic polymorphisms vary widely [8].

Glutathione-S-transferase P1 (*GSTP1*) is a rate-limiting enzyme that catalyzes the detoxification pathway of platinum drugs, for cell protection. The *GSTP1* Ile105Val (rs1695, A>G) is a missense mutation and affects the activity of the *GSTP1* enzyme. Oxaliplatin-related cumulative peripheral neuropathy was reported to be more frequent and severe in patients with heterozygous (AG) genotype when compared to patients with wild-type alleles (AA) [9]. Wiese et al. [10] conclude that *GSTP1* c.313A>G polymorphism was associated with the overall survival of patients treated with oxaliplatin-based therapy. From the above, we hypothesize that *DPYD* and *GSTP1* polymorphisms could be linked to acute toxicity in patients undergoing 5-FU and oxaliplatin-based chemotherapy.

Our study aims to board the possible relationship between *DPYD* and *GSTP1* genomic variations and adverse reactions with 5-FU and oxaliplatin-based treatment in the Meizhou Hakka population, southern China, a unique Han Chinese ethnic group having its civilization [11]. We performed a systematic retrospective pharmacokinetics study in a cohort of 104 Meizhou Hakka CRC patients for the first time to explore the associations.

## Patients and methods

### Population study and data collection

One hundred and four registered patients with histologically confirmed CRC who received adjuvant chemotherapy regimen containing 5-FU, capecitabine, or oxaliplatin between September 2016 and December 2017 at the Meizhou People’s Hospital were investigated in this retrospective study. Patients had to follow the lists for inclusion criteria such as (1) histopathologically proven colorectal cancers in any stage; (2) aged over 18 years; (3) patients received adjuvant chemotherapy containing 5-FU, capecitabine, or oxaliplatin in any line of treatment and received at least 2 courses for treatment; (4) any previous chemotherapy completed half a year ago, without any other adjuvant chemotherapy or radiotherapy; (5) life expectancy > 4 months; (6) adequate hematological, liver, or kidney functions. Patients were excluded from enrollment as follows: (1) patients with concomitant neoplastic disease other than colorectal cancer; (2) any non-compliance of the inclusion criteria of above; (3) severe renal or hepatic disorder, severe hematological disease before treatment; (4) less than 6 months of previous treatment unless appear greater than grade 3 adverse events. Patients who disagree to sign an informed consent form were excluded from the research. Written informed consent was provided for each patient before any procedure of this study. All participants were then followed up 18 months to obtain their detailed clinical response information and recorded in a medical history sheet. The study protocol was approved by the institution’s review and Human Ethics Committees of the Meizhou Peoples’ Hospital (No. MPH-HEC 2016-A-40). Clinical characteristics distribution of 55 men and 49 women newly diagnosed with CRC from 2016 to 2017 were enrolled in the final study. The demographic and clinical-pathological features of our study cohort were evaluated as summarized in Table 1. The median age at diagnosis was 56 (range 25–78). All adverse drug reactions and toxicity underwent in the front 6 cycles of chemotherapy in each patient were monitored and graded in line with the National Cancer Institute Common Terminology Criteria for Adverse Events v3.0 (CTCAE) [12].

**Table 1:** Demographic and clinical characteristics of treated patients

Characteristics	Overall patients, *n* (%)	Adverse events	
		G1–2, *n*	G3–4, *n*
Age (years)			
Range	25-78	45–74	25–78
Median	56	61.5	53
≤ 56	50(48.1)	9	44
> 56	54(51.9)	17	29
Gender			
Male	55(52.8)	19	34
Female	49(47.1)	7	39
Smoking status			
Smoker	8(7.6)	2	5
Non-smoker	96(92.3)	24	68
Alcohol intake			
Yes	4(3.8)	2	2
No	100(96.1)	24	71
Anatomic site			
Rectum	39(37.5)	11	27
Colon	65(62.5)	15	46
Histopathological type			
Adenocarcinoma	104(100.0)	26	73
Squamous cell carcinoma	0(0)	0	0
Adenocarcinoma histology			
Poorly differentiated	1(1)	0	1
Moderately differentiated	57(54.8)	13	42
Mucinous adenocarcinoma	3(2.9)	0	3
Undefined	43(41.3)	13	27
Grade of tumor			
I	0(0)	0	0
II	30(28.8)	6	22
III	46(44.2)	15	31
IV	28(27)	5	20
Clinical T-stage			
1	2(1.9)	0	2
2	3(2.9)	1	2
3	65(62.5)	19	44
4	34(32.7)	6	25
Clinical N-stage			
0	33(31.7)	7	24
1	42(40.4)	12	30
2	28(26.9)	7	18
3	1(1)	0	1
Clinical M-stage			
0	76(73)	21	53
1	28(27)	5	20

### Genotyping

All collected blood samples were anticoagulants with EDTA. Genomic DNA from each enrolled patient was extracted using the DNA Isolation Kit for Blood/Bone Marrow/Tissue (Tiangen Biotech (Beijing) Co., Ltd.). DNA concentration and purity were evaluated using a NanoDrop2000 (Thermo Scientific). The primer sequences and the restriction enzymes used were provided by SINOMD Gene (Beijing) Co., Ltd. Genomic DNA was used for the cyclic condition consisted of one predenaturation step at 95 °C for 3 min initial cycle, then 45 cycles of 94 °C for 15 s, 63 °C for 1 min and extension at 72 °C for 1 min, and a final elongation step of 10 min at 72 °C. PCR products were purified with ExoSap-It (ABI PCR Product Cleanup Reagent). Single-nucleotide polymorphisms were analyzed using ABI 3500 Dx Genetic Analyzer (ABI Terminator v3.1 Cycle Sequencing kit) and Sequencing Analysis v5.4 (Life Technologies, CA, USA). We looked for the following SNPs: DPYD*2A, DPYD*5A, DPYD*9A, and *GSTP1*.

### Toxicity grading and statistical analysis

All quantitative variables such as age, gender, smoking, alcohol intake, tumor location, neoplasms histologic type, grade, and stage of the tumor were analyzed. Toxicity including hematopoietic toxicity (anemia, leucopenia, neutropenia, and thrombocytopenia), gastrointestinal toxicity (mucositis, vomiting, and diarrhea), and dermatologic toxicity (ulceration and hand-foot syndrome) were grouped by four classes. Association between *DPYD* and *GSTP1* variant status with a response and different toxicity was evaluated by Fisher’s exact test. The Hardy-Weinberg equilibrium (HWE) of *DPYD* and *GSTP1* genotypes were assessed using the *χ*^2^ test. A *p* value of less than 0.05 was deemed to be statistically significant. All tests were carried out by the SPSS software version 21.0 (SPSS Inc., Chicago, USA)

## Results

### Histopathological type and clinical stage

As shown in Table 1, colon cancer was more predominant than rectum cancer (62.54 and 37.5%, respectively) about tumor location. All histopathological types were adenocarcinoma. Seventy-four (46.9%) patients were affected by stage III–IV tumors. Following the American Joint Committee on Cancer’s (AJCC) Cancer Staging 6th edition 2002 TNM grading systems, there were 28.8% of stage II, 44.2% of stage III, and 27% of stage IV about the grade of the tumor, but none of stage I.

### Chemotherapy toxicity

The toxicity of chemotherapy in the study participants is shown in Table 2. Treatment of fluoropyrimidine and platinum combination chemotherapy was made at the time of the first diagnosis of CRC. All patients received chemotherapy regimens for at least 2 cycles and were evaluated for adverse event outcomes. Of the total 104 cases, 72 patients (69.2%) develop severe chemotherapy-related toxicities, including 18.3% suffered from anemia, 9.6% for leukopenia, 15.4% for neutropenia, 11.5% for thrombocytopenia, 8.7% for mucositis, 8.7% for diarrhea, 22.1% for vomiting, and 8.6% for dermatological toxicities.

**Table 2:** Toxicity of chemotherapy in the study participants

Toxicities	Grade 1–2 *n* (%)	Grade 3–4 *n* (%)
Hematological toxicity		
Anemia	46(44.2)	19(18.3)
Leucopenia	48(46.2)	10(9.6)
Neutropenia	39(37.5)	16(15.4)
Thrombocytopenia	39(37.5)	12(11.5)
Gastrointestinal toxicity		
Mucositis	4(3.8)	9(8.7)
Vomiting	27(25.9)	23(22.1)
Diarrhea	12(11.0)	9(8.7)
Dermatology toxicity		
Skin ulceration	5(4.8)	4(3.8)
Hand-foot skin reaction	11(10.6)	5(4.8)

### Dihydropyrimidine dehydrogenase and glutathione S-transferase pi-1 genotypes

In our cohort, all patients were genotyped for DPYD*2A, DPYD*5A, DPYD*9A, and *GSTP1* variants. The *DPYD* [IVS] 14+1G>A mutation was not identified in any of this study patients. In the case of DPYD*5A polymorphism, *DPYD* c. 1627A>G was present in 33 (31.7%) patients with heterozygous and 7 (6.7%) in homozygous, and there were 76% wild types and 24% heterozygote mutants to *DPYD* c. 85T>C. The results also show that the distribution of *GSTP1* A/A, *GSTP1* A/G, and *GSTP1* G/G was 67.3%, 29.8%, and 2.9%, respectively (Table 3). Determined SNPs were found to be in Hardy-Weinberg Equilibrium.

**Table 3:** DPYD and GSTP1 genes frequency in the study patients

Gene	Genotypes	No. of patients	Percentage (%)
DPYD*2A	WT(GG)	104	100.0
	HET(GA)	0	0.0
	HOM(AA)	0	0.0
DPYD*5A	WT(AA)	64	61.5
	HET(AG)	33	31.7
	HOM(GG)	7	6.7
DPYD*9A	WT(TT)	79	76.0
	HET(TC)	25	24.0
	HOM(CC)	0	0.0
GSTP1	WT(AA)	70	67.3
	HET(AG)	31	29.8
	HOM(GG)	3	2.9

*WT* = wild type, *HET* = heterozygote, *HOM* = homozygote

### Adverse events

The distribution of toxicity classification of patients with *DPYD* and *GSTP1* wild type and mutant was shown in Tables 4 and 5, respectively. Among patients screened for *DPYD* mutant (*n* = 56), 40 patients had *DPYD* c. 1627A>G (DPYD*5A) variant, while *DPYD* c. 85T>C (DPYD*9A) variant exists in 25 patients. Out of them in 9 patients with both DPYD*5A and DPYD*9A heterozygous. The most commonly experienced grade 3 or grade 4 toxicity in both *DPYD* wild-type and mutant patients was anemia in hematological and vomiting in gastrointestinal toxicity (Table 4). The same results were found in patients with *GSTP1* wild type and mutants (Table 5).

**Table 4:** The frequency of all grade toxicities in patients with *DPYD* wild type and mutants

	DPYD wild type (*n* = 48)		DPYD mutant (*n* = 56)			
			DPYD*5A (*n* = 40^*^)		DPYD*9A (*n* = 25^*^)	
Toxicities	G 1–2	G 3–4	G 1–2	G 3–4	G 1–2	G 3–4
Hematological toxicity						
Anemia	22(21.2)	9(8.7)	17(16.3)	8(7.7)	12(11.5)	4(3.8)
Leucopenia	21(20.2)	4(3.8)	20(19.2)	4(3.8)	11(10.6)	3(2.9)
Neutropenia	17(16.3)	5(4.8)	17(16.3))	8(7.7)	8(7.7)	6(5.8)
Thrombocytopenia	16(15.4)	4(3.8)	16(15.4)	6(5.8)	12(11.5)	4(3.8)
Gastrointestinal toxicity						
Mucositis	2(1.9)	7(6.7)	2(1.9)	2(1.9)	0(0)	0(0)
Vomiting	12(11.5)	13(12.5)	13(12.5)	7(6.7)	5(4.8)	4(3.8)
Diarrhea	7(6.7)	7(6.7)	5(4.8)	2(1.9)	1 (0.9)	1(0.9)
Dermatology toxicity						
Skin ulceration	1(0.9)	1(0.9)	4(3.8)	3(2.9)	0(0)	0(0)
Hand-foot skin reaction	4(3.8)	1(0.9)	6(5.8)	4(3.8)	1(0.9)	0(0)

Data are presented as *n* (%)

*9 patients had double heterozygous status for DPYD*5A and DPYD*9A variants

**Table 5:** The frequency of all grade toxicities in patients with *GSTP1* wild type and mutants

	GSTP1 wild type (*n* = 70)		GSTP1 mutant (*n* = 34)	
Toxicities	G 1–2	G 3–4	G 1–2	G 3–4
Hematological toxicity				
Anemia	31(29.8)	14(13.5)	15(14.4)	5(4.8)
Leucopenia	35(33.7)	7(6.7)	13(12.5)	3(2.9)
Neutropenia	30(28.8)	13(12.5)	9(8.7)	3(2.9)
Thrombocytopenia	26(25.0)	8(7.7)	13(12.5)	4(3.8)
Gastrointestinal toxicity				
Mucositis	2(1.9)	7(6.7)	2(1.9)	2(1.9)
Vomiting	22(21.2)	12(11.5)	5(4.8)	11(10.6)
Diarrhea	8(7.7)	7(6.7)	4(3.8)	2(1.9)
Dermatology toxicity				
Skin ulceration	5(4.8)	1(0.9)	0(0)	3(2.9)
Hand-foot skin reaction	8(7.7)	2(1.9)	3(2.9)	3(2.9)

Data are presented as *n* (%)

### Association between SNPs and toxicities

Association between polymorphisms of *DPYD* and *GSTP1* genes and toxicities of chemotherapy outline is shown in Tables 6, 7, and 8. Adverse events were experienced in most patients who received chemotherapy after the first or second cycle. We further examined the association between these SNPs and adverse events in all individuals. The existence of the *GSTP1* c. 313A>G polymorphism was associated with platinum related to several toxicities such as vomiting and skin ulceration (*p* = 0.042 and *p* = 0.018, respectively). We also found that patients with the AA genotype for *GSTP1* presented lower rates of serious vomiting (35.3%) than patients with the AG and GG genotype (66.7% and 100%, respectively, *p* = 0.027). Hand-foot syndrome and hematopoietic toxicity could not be demonstrated a statistically significant association with *GSTP1* genotype in our study. However, 5-FU and capecitabine-related toxicities were not correlated to *DPYD* polymorphism, although there was a statistical trend for more diarrhea in the patients when carrying the mutant DPYD*5A A/G genotype.

**Table 6:** Association between polymorphisms of *DPYD* and *GSTP1* genes and hematological toxicity of chemotherapy

Genotype		Anemia			Neutropenia			Thrombocytopenia			Leucopenia		
		Grade 1–2	Grade 3–4	*P*	Grade 1–2	Grade 3–4	*P*	Grade 1–2	Grade 3–4	*P*	Grade 1–2	Grade 3–4	*P*
DPYD*5A (c.1627A > G)	WT	29	11	Ref.	22	8	Ref.	23	6	Ref.	28	6	Ref.
	HET	16	7	0.804	14	7	0.607	14	5	0.650	17	4	0.896
	HOM	1	1	0.492	3	1	0.943	2	1	0.614	3	0	0.427
	HET + HOM	17	8	0.698	17	8	0.665	16	6	0.583	20	4	0.922
DPYD*9A (c.85T>C)	WT	34	15	Ref.	31	10	Ref.	27	8	Ref.	37	7	Ref.
	HET	12	4	0.668	8	6	0.189	12	4	0.867	11	3	0.794
	HOM	0	0	–	0	0	–	0	0	–	0	0	–
	HET + HOM	12	4	0.668	8	6	0.189	12	4	0.867	11	3	0.794
GSTP1 (c.313A>G)	WT	31	14	Ref.	30	13	Ref.	26	8	Ref.	35	7	Ref.
	HET	14	4	0.480	9	3	0.724	11	3	0.875	11	2	0.913
	HOM	1	1	0.575	0	0	–	1	1	0.401	2	1	0.466
	HET + HOM	15	5	0.617	9	3	0.724	12	4	0.910	13	3	0.851

**Table 7:** Association between polymorphisms of *DPYD* and *GSTP1* genes and gastrointestinal toxicity of chemotherapy

Genotype		Mucositis			Vomiting			Diarrhea		
		Grade 1–2	Grade 3–4	*P*	Grade 1–2	Grade 3–4	*P*	Grade 1–2	Grade 3–4	*P*
DPYD*5A (c.1627A>G)	WT	2	7	Ref.	14	16	Ref.	7	7	Ref.
	HET	1	2	0.700	12	5	0.113	4	2	0.052
	HOM	1	0	0.107	2	2	0.900	1	0	0.533
	HET + HOM	2	2	0.317	14	7	0.158	5	2	0.107
DPYD*9A (c.85T>C)	WT	4	9	Ref.	23	19	Ref.	11	8	Ref.
	HET	0	0	–	5	4	0.965	1	1	0.830
	HOM	0	0	–	0	0	–	0	0	–
	HET + HOM	0	0	–	5	4	0.965	1	1	0.830
GSTP1 (c.313A>G)	WT	2	7	Ref.	22	12	Ref.	8	7	Ref.
	HET	2	2	0.317	5	10	**0.042**	4	2	0.577
	HOM	0	1	0.598	0	1	0.187	0	0	–
	HET + HOM	2	3	0.480	5	11	**0.027**	4	2	0.577

**Table 8:** Association between polymorphisms of *DPYD* and *GSTP1* genes and dermatology toxicity of chemotherapy

Genotype		Skin ulceration			Hand-foot skin reaction		
		Grade 1–2	Grade 3–4	*P*	Grade 1–2	Grade 3–4	*P*
DPYD*5A (c.1627A>G)	WT	2	1	Ref.	5	1	Ref.
	HET	2	1	1.000	4	3	0.308
	HOM	2	2	0.659	2	1	0.571
	HET + HOM	4	3	0.778	6	4	0330
DPYD*9A (c.85T>C)	WT	6	4	Ref.	10	5	Ref.
	HET	0	0	–	1	0	0.486
	HOM	0	0	–	0	0	–
	HET + HOM	0	0	–	1	0	0.486
GSTP1 (c.313A>G)	WT	5	1	Ref.	8	2	Ref.
	HET	0	3	**0.018**	3	3	0.210
	HOM	0	0	–	0	0	–
	HET + HOM	0	3	**0.018**	3	3	0.210

## Discussion

Fluorouracil (FU) is the most common component of chemotherapy agents for malignant tumors like leukemia, lung, breast, gastric, and colorectal cancers. Falzacappa et al. demonstrated combined fluorouracil, and rucaparib was able to enhance the efficacy and specificity of acute myeloid leukemia [13]. Adrian BogdanTigu et al. highlighted that the combination of allicin with 5-FU has an obvious against lung and colorectal cancer effect while reducing the dosage of fluorouracil [14]. For breast cancer, fluorouracil (5-FU) is widely used and a growing number of physicians are using capecitabine as first-line or second-line chemotherapy [15]. Adjuvant chemoradiotherapy based on 5-fluorouracil is the first choice after gastric cancer surgery. Li et al. suggested that accurate classification of GC patients who may benefit from 5-FU adjuvant chemoradiotherapy will help clinicians formulate more effective GC treatment regimens [16].

Fluorouracil-based treatment is generally well tolerated, except about 5 to 10% patient who suffers grade > 3 toxicity, which even impacts on the quality of life [17]. Intolerance of 5-FU is usually because of decreased activity of the fluoropyrimidine detoxifying enzyme dihydropyrimidine dehydrogenase (DPD) [18]. DPD is known to be the initial and rate-limiting enzyme catalyze degradation of more than 85% of the given 5-FU. Activity levels vary caused by variable phenotypes and genetic alterations [19]. Single-nucleotide polymorphisms (SNPs) in its encoding gene *DPYD* is the most common cause of deficiency in DPD enzyme activity [20]. Analyzing the impact of *DPYD* genotype in serum levels of 5-FU and correlative toxicity is vital to achieving good curative effect and prevent adverse events. In this research, we looked for adverse events related to fluoropyrimidines and platinum drugs-based treatment and their association with *DPYD* and *GSTP1* gene polymorphisms. Vomiting and skin ulceration was the most chemotherapy-related toxicity observed in our study.

The allelic variant (A>G) at position 1627 amino acid in exon 13 of the *DPYD* gene results in an exchange of Isoleucine to Valine at codon 543 of DPYD*5A. There is a wide interethnic and inter geographical difference about gene polymorphism and frequency of DPYD*5A. DPYD*5A G/G + G/A genotype was identified in 38.4% participants, obviously more than that in African-Americans (22.7%) [21] and Caucasian subjects (28%) [22], and similar to that in Japanese (35%) [23]. DPYD*9A polymorphism is regarded as one prevalent *DPYD* variants. The TT and TC genotype proportion of DPYD*9A in our enrolled patients was 76% and 24%, respectively, whereas the homozygous DPYD*9A genotype was not found. The effect of DPYD*9A polymorphism on fluoropyrimidine treatment-related toxicity has been reported in previous researches. Khushman et al. [24] showed that *DPYD* c. T85C mutant variant was associated with diarrhea (*p* = 0.0055). Joerger [25] reported that the subjects carrying the DPYD*9A C allele significantly increase the risk for severe diarrhea compared to patients with the T/T genotype (*p* = 0.033). Nevertheless, several other studies suggest different points of view. Maarten [26], Amirfallah [27], and McLeod et al. [28] revealed that *DPYD* heterozygous for 85T>C was not associated with a decreased risk for grade 3 to 4 toxicity. In our study cohort, we haven't found a statistically significant relationship between DPYD*2A, *5A, *9A polymorphism and severe chemotherapy-related toxicity, owing to the limited size of the cohort in our study, and different allele frequency of these gene variants among different populations in different regions.

As a part of the phase II detoxification system, glutathione S-transferase (GST) superfamily members detoxifies cisplatin by the formation of glutathione conjugates, conversion to less toxic and increases its excretion from the body, resulting in the detoxification [29]. It is important to note that polymorphisms in GSTs are linked to the occurrence and development of various tumors by way of altering biological pathways and affecting protein expression [30]. Genes encoding GSTs, including *GSTP1*, different genotypes have different effects on the efficacy to detoxify exogenous and endogenous toxic species. Extensive research has been carried out to reveal the 313A>G (*GSTP1*) mutation is an A to G replacement at codon 105 leading to a transition in the amino acid from Isoleucine to Valine, which has been found to decrease enzymatic activity, and highly linked for causing platinum treatment-related toxicity in different cancers.

In previous studies, Santric [31] reported a significant association between *GSTP1* Ile105Val polymorphism and oxaliplatin-related toxicity. Kudhair [32] analyzed Arab populations and revealed that carrying *GSTP1* Ile105Val substitution is positively correlated with a higher risk for lung cancer in WP tobacco smokers. Conversely, Sophonnithiprasert [33] reported an insignificant difference between *GSTP1* Ile105Val polymorphism and oxaliplatin-induced toxicity. Our research showed that *GSTP1* c. 313A>G polymorphism was linked to acute toxicity in colorectal cancer patients undergoing skin ulceration, which was conflicting with research from Terrazzino [34] and Osti [35]. We also found that those participants having heterozygotes and homozygotes genotype showed a higher risk of high-grade severe toxicity compared to the wild type of *GSTP1*, in line with the reported study of Mir [36]. According to the research by Watson, it was suggested that GST enzyme activities were significantly reduced among individuals with Val-containing [37]. Besides, there was lower activity toward chemotherapy drug tolerance [38]. As mentioned above, the *GSTP1* c.313 A > G mutation leads to a decrease in enzyme activity, thereby reducing the ability to detoxify platinum and other substances. Skin ulceration may be a direct result of the transport of the residual oxaliplatin active substance to the skin surface via sweat. Oxaliplatin results in the formation of intra and inter strands crosslinks, causing potential errors in DNA repair and damaged DNA. Finally, it diminishes numbers of endothelial cells and dermal matrix regeneration, leading to the occurrence and development of skin ulceration.

In Table 7, we found an interesting association between *GSTP1* A/G genotype and severe vomiting in patients (*p* = 0.042), which consistent with Carron et al. [39]. As the detoxification ability of GSTP1 A/G genotype decreases, platinum accumulates in the body causes vomiting by inducing DNA damage in epithelial enterochromaffin cells of the intestine, thereby leading to serotonin release and stimulation of the vomiting center.

There are some limitations to our study. First, the number of subjects enrolling in the research is relatively small. Second, we did not investigate other SNPs of GST associated with oxaliplatin-related toxicities. The third limitation of this study is that we focus on only three out of more than 30,000 variants reported *DPYD* variants. Moreover, there is a difference in chemotherapy regimens. Future analysis will enroll a larger cohort and reveal the potential associations between more mutation combinations of *DPYD* and GST variants and severe toxicities.

## Conclusions

In brief, skin ulceration and vomiting were the major dose-limiting toxicity of fluoropyrimidine and platinum-based regimens in this cohort. A significant correlation was confirmed between *GSTP1* polymorphism and toxicity like vomiting and skin ulceration strengthening *GSTP1* polymorphism as a predictor of severe events. Screening of genetic polymorphism of *GSTP1* in colorectal cancer patients before chemotherapy could contribute to effectively decrease the occurrence of serious adverse toxicity, improve the predictive specificity for a severe event. We indicate that the detection of *GSTP1* phenotype may become a routine laboratory test item. This may enable clinicians to implement the optimal therapeutic schedule to achieve satisfactory efficacy and minimize toxic and side effects, and then, lead to personalized therapy. Further studies should focus on validating results in a larger sample size, ascertain possible interactions between other *DPYD* and GST polymorphisms, and clinical outcomes.

## Data Availability

Not applicable

## Abbreviations

CRC: Colorectal cancer
*DPYD*: Dihydropyrimidine dehydrogenase
*GSTP1*: Glutathione S-transferase pi-1
SNP: Single-nucleotide polymorphisms
